# Neuropilin-1 Expression on Regulatory T Cells Enhances Their Interactions with Dendritic Cells during Antigen Recognition

**DOI:** 10.1016/j.immuni.2008.01.012

**Published:** 2008-03-14

**Authors:** Milka Sarris, Kristian G. Andersen, Felix Randow, Luzia Mayr, Alexander G. Betz

**Affiliations:** 1 Medical Research Council, Laboratory of Molecular Biology, Hills Road, Cambridge CB2 0QH, UK

**Keywords:** MOLIMMUNO, CELLIMMUNO

## Abstract

The interaction of T cells with dendritic cells (DCs) determines whether an immune response is launched or not. Recognition of antigen leads to formation of immunological synapses at the interface between the cells. The length of interaction is likely to determine the functional outcome, because it limits the number of MHC class II-peptide complexes that can be recruited into the synapse. Here, we show that regulatory T (Treg) cells and naive helper T (Th) cells interact differently with DCs in the absence of proinflammatory stimuli. Although differences in T cell receptor repertoire might contribute, Foxp3-induced phenotypic differences play a major role. We found that Neuropilin-1 (Nrp-1), which is expressed by most Treg cells but not naive Th cells, promoted prolonged interactions with immature DCs (iDCs), resulting in higher sensitivity to limiting amounts of antigen. This is likely to give Treg cells an advantage over naive Th cells, with the same specificity leading to a “default” suppression of immune responses in the absence of “danger signals.”

## Introduction

The interaction between helper T (Th) cells and dendritic cells (DCs) has been studied extensively. The contact zone between DCs and T cells is often referred to as immunological synapse (Friedl et al., 2005). The molecular interactions within these synapses are dynamic, revealing different patterns of interactions between surface molecules depending on the presence or absence of antigen, the activation status of T cells and DCs, and the temporal stage of the immune response (Friedl et al., 2005). During an interaction, T cell receptor (TCR) molecules are recruited to the central area of the synapse into central supramolecular activation clusters (cSMACs), whereas several adhesion molecules accumulate in the peripheral area of the synapse (pSMAC) (Monks et al., 1998). TCR molecules initially form microclusters, which converge to form the cSMAC (Krummel et al., 2000). It has been proposed that TCR microclusters, which continuously form in the periphery, initiate and sustain signaling, whereas their movement to the cSMAC is associated with TCR degradation (Varma et al., 2006; Yokosuka et al., 2005). The dynamics and molecular composition of synapses between DCs and regulatory T (Treg) cells remain uncharacterized.

Treg cells play an important role in the prevention of autoimmunity (Sakaguchi, 2005), maternal-fetal tolerance (Aluvihare et al., 2004), and other immune responses that are legitimate in principle but potentially detrimental to the organism (Sakaguchi, 2005). Treg cells were originally characterized on the basis of the presence of cell-surface markers such as CD4 and CD25. Since then, expression of the lineage marker Foxp3 has become the accepted gold standard for the identification of natural Treg cells (Sakaguchi, 2005). Once activated, Treg cells suppress the activation and function of Th cells, B cells, and DCs (Miyara and Sakaguchi, 2007). Although it is apparent that interaction of Treg cells with DCs is required for their activation under physiological conditions (Tarbell et al., 2006), the mechanistic details remain elusive.

Experimental removal of Treg cells (Sakaguchi et al., 1995; Kim et al., 2007) or interference with their recruitment and/or retention (Bystry et al., 2001) leads to the rapid launch of autoimmune responses in unprimed hosts. This suggests that autoreactive Th cells are constitutively present. In this context, the decision as to whether to launch an autoimmune response appears to be fought out between Treg cells and naive Th cells, with the Treg cells having the upper hand in a healthy animal.

Here, we show that Treg and naive Th cell populations interact differently with immature DCs (iDCs). Although differences in the respective T cell receptor repertoires are likely contributors, they are not the sole component. Ectopic expression of Foxp3 leads to a marked change in interaction behavior. Foxp3 modulates a multitude of genes (Hill et al., 2007), and we demonstrated that one of them, Neuropilin-1 (Nrp-1), which is expressed by Treg cells but not naive Th cells (Bruder et al., 2004), played a key role in promoting long interactions between Treg cells and iDCs. Whereas blocking of Nrp-1 decreased the frequency of long interactions, ectopic expression of Nrp-1 in Th cells increased the number of long interactions, synapse formation, and the sensitivity of the cells to their cognate antigen.

## Results

### Treg Cells Make Frequent MHC Class II-Dependent Long Interactions with iDCs

**Figure 1:**
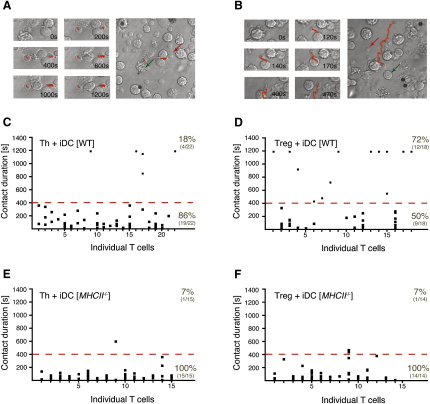
Treg Cells Form More MHC Class II-Dependent Long Interactions with iDCs Than Naive Th Cells CD4^+^CD25^+^ (Treg) or CD4^+^CD25^−^ (Th) cells were cocultured with iDCs and imaged as described in the Experimental Procedures. (A and B) Representative examples of T cells forming either (A) long interactions or (B) multiple short interactions with iDCs. Snapshots of the area surrounding the traced T cell at the indicated time points are shown (left). The complete path (red trace) traversed by the T cell in 20 min is shown (right). Representative T cells (red arrows) and iDCs (green arrows) have been marked. (C–F) Interactions observed between (C and E) Th or (D and F) Treg cells and (C and D) WT or (E and F) MHC class II-deficient iDCs (*MHCII*^−/−^) in individual experiments. Columns represent T cells with each of the dots denoting the length of an interaction made. All T cells that have made at least one contact with an iDC are included. The frequency of T cells interacting with an iDC for longer or shorter than 400 s (dashed red line) is given as percentage and as ratio.

**Figure 2:**
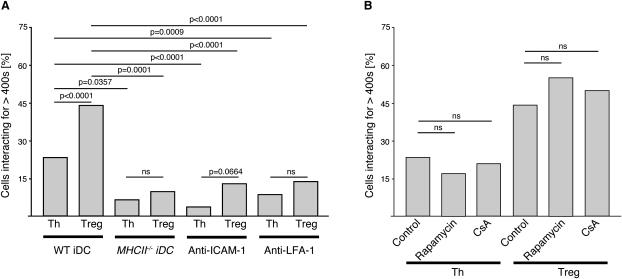
Long Interactions with iDCs Are ICAM-1 and LFA-1 Dependent Percentage of CD4^+^CD25^+^ (Treg) and CD4^+^CD25^−^ (Th) cells forming long interactions (>400 s) with iDCs among all interacting cells is shown. (A) Interactions of Treg or Th cells with WT iDC (WT iDC; eight independent experiments, n = 185 for Treg cells and n = 264 for Th cells), MHC classII-deficient iDC (*MHCII*^−/−^ iDC; two independent experiments, n = 31 for either Treg and Th cells), WT iDC in the presence of anti-ICAM-1 (two independent experiments, n = 70 for Treg cells and n = 82 for Th cells), and WT iDC in the presence of anti-LFA-1 (two independent experiments, n = 85 for Treg cells and n = 113 for Th cells). (B) Interactions of Treg or Th cells with WT iDCs in the presence of the inhibitors rapamycin (n = 29 for Th cells and n = 47 for Treg cells) and CSA (n = 28 for Th cells and n = 18 for Treg cells) or absence of inhibitors (eight independent experiments, n = 264 for Th cells and n = 185 for Treg cells). Relevant p values (Fischer's exact test) are given. ns stands for statistically not significant.

Although interactions between DCs and Th cells have been visualized in real time both in vivo and ex vivo (Benvenuti et al., 2004; Miller et al., 2004b; Shakhar et al., 2005), data on the dynamics of the interactions between DCs and Treg cells are scarce (Tadokoro et al., 2006; Tang et al., 2006). To investigate potential differences in the behavior of Treg and Th cells, we used time-lapse video microscopy (Movies S1 and S2 available online). All studies were performed with bone-marrow-derived DCs, which are representative of nonactivated iDCs (Wan and Dupasquier, 2005). Freshly isolated CD4^+^CD25^+^ Treg or CD4^+^CD25^−^ Th cells were cocultured with iDCs and monitored for the first 20 min of their coculture. In either case, we observed short and long interactions between the T cells and the iDCs (Figures 1A and 1B). Transient contacts between T cells and antigen-presenting cells (APCs) are characteristic of T cell scanning for antigen (Irvine et al., 2002; Miller et al., 2004a). Because we observed the formation of long interactions between both CD4^+^CD25^−^ Th and CD4^+^CD25^+^ Treg cells with iDCs in the absence of exogenous antigen (Figures 1C and 1D), we wanted to address whether they are dependent on the presence of MHC class II-endogenous peptide complexes. In either case, virtually all interactions with iDCs prepared from MHC class II *H2-Ab1* gene-deletion mutant mice (Grusby et al., 1991) lasted less than 400 s (Figures 1E, 1F, and 2A; Movie S3). In contrast, a considerable number of interactions of CD4^+^CD25^+^ or CD4^+^CD25^−^ cells with wild-type (WT) iDCs exceeded 400 s (Figures 1C, 1D, and 2A). Thus, recognition of MHC class II-peptide complexes is a crucial prerequisite for long interactions (>400 s), providing us with a rational threshold for an extended analysis of our data. The frequency of CD4^+^CD25^+^ cells forming long interactions with iDCs (Figure 2A) was almost twice as high as that of the CD4^+^CD25^−^ cells (44.3% versus 23.5% of all cells forming interactions, p < 0.0001, Fisher's exact test). In an unprimed host, the vast majority of the CD4^+^CD25^−^ cells are naive, antigen-inexperienced Th cells, and the vast majority of CD4^+^CD25^+^ cells are Treg cells. The frequency of memory- or effector-type cells contained in the CD4^+^CD25^−^ cell populations is too low to substantially skew the results. Therefore, we conclude that the differences observed are largely due to the differences in the interaction behavior of Treg and naive Th cells.

### Long Interactions Only Require Proximal TCR Signaling

Recognition of MHC class II-peptide complexes locks LFA-1 into its high-affinity conformation (Dustin et al., 1997), thereby increasing interaction with ICAM-1 on the DC. This stop signal stabilizes the interaction between T cells and APCs. The presence of either anti-ICAM-1 (Movie S4) or anti-LFA-1 led to a significant drop in long interactions of both CD4^+^CD25^+^ and CD4^+^CD25^−^ cells with WT iDCs (Figure 2A), corroborating the role of MHC class II-peptide recognition in the establishment and/or maintenance of long contacts.

The length of interaction between T cells and DCs is influenced by TCR signaling events. TCR signaling events such as Ca^2+^ influx reinforce the “stop signal” mediated by ICAM-1 and LFA-1 (Dustin et al., 1997) and are required for stable interactions between CD4^+^ T cells and B cells, whereas late TCR signaling events such as activation of calcineurin are not required (Negulescu et al., 1996). Pretreatment of the T cells with either rapamycin, which blocks Ca^2+^-independent signaling pathways, or the calcineurin inhibitor cyclosporin-A (CsA) (Gerber et al., 1998) did not significantly affect the frequency of long interactions with iDCs (Figure 2B), suggesting that this process does not depend on distal TCR signaling events.

### Ectopic Expression of Foxp3 in Th Cells Prolongs Their Interactions with iDCs

**Figure 3:**
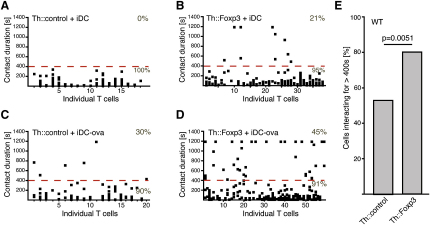
Increased Frequency of Long Interactions upon Ectopic Expression of Foxp3 (A–D) Interactions between (A and C) Th::control and (B and D) Th::Foxp3 cells prepared from DO11.10x*Rag2*^−/−^ mice and (A and B) untreated iDCs or (C and D) ova-loaded iDCs (100 μg/ml; 12 hr) analyzed as outlined in Figure 1. (E) Summary of the interactions observed in coculturing experiments of either WT Th::control or WT Th::Foxp3 cells with iDCs in the absence of exogenous antigen (three independent experiments, n = 56 for Th::Foxp3 cells and n = 45 for Th::control cells; p = 0.0051, Fischer's exact test). The percentage of cells forming long interactions (>400 s) among all interacting cells is shown.

Initially, we assumed that the difference in interaction behavior can be attributed to differences in the frequency of self-reactive cells in the repertoire of Treg cells versus that of Th cells (Hsieh et al., 2004). However, when we ectopically expressed Foxp3 in DO11.10 × *Rag2*^−/−^ CD4^+^ T cells to obtain Foxp3-induced regulatory T cells expressing a TCR specific for ovalbumin (ova) (DO11.10-Th::Foxp3), we found that they form substantially more long interactions than those transduced with a control gene (DO11.10-Th::control cells) (Figure 3 and Figure S1A). The amount of retrovirally expressed Foxp3 was only marginally higher (both mRNA and protein) than the endogenous amounts found in CD4^+^CD25^+^ Treg cells (Figures S1B and S1C). To confirm the conversion of Th cells toward a Treg cell-like phenotype, we examined the Th::Foxp3 cells for the expression of CD25, Lag3, and GITR (Figures S1F–S1H) and found them to be upregulated in these cells. In contrast to Th::control cells, the Th::Foxp3 cells were anergic (Figure S1D) and suppressed the proliferation of CD4^+^CD25^−^ Th cells (Figure S1E). In the absence of ova, none of the Th::control cells formed long interactions (Figure 3A), whereas 21% of the Th::Foxp3 cells formed long interactions with iDCs (Figure 3B). When the iDCs were loaded with ova, 30% of Th::control cells formed long interactions (Figure 3C). This increase in long interactions was more marked for Th::Foxp3 cells (Figure 3D), suggesting that Foxp3-induced phenotypic differences between Treg cells and naive Th cells contribute to differences in the dynamics of the cell-cell interactions.

To address the degree to which Foxp3-induced factors contribute to differences in interaction behavior between Treg and naive Th cells, we compared WT Th::Foxp3 and Th::control cells. The transduction process in itself led to the CD4^+^CD25^−^ cells forming more long interactions (53.3%; Figure 3E) than freshly isolated CD4^+^CD25^−^ cells (23.5%; Figure 2). This was most likely due to a change in the activation status of the cells (Rothoeft et al., 2006), because activation of the cells is required to allow retroviral transduction. However, Th::Foxp3 cells formed significantly more long interactions (80.4%) than Th::control cells (53.3%) (Figure 3E). Qualitatively, this difference was similar to that observed between freshly isolated CD4^+^CD25^−^ Th and CD4^+^CD25^+^ Treg cells. Although differences in the TCR repertoire between Treg and Th cells are likely to account for part of the differences in the interaction behavior, this demonstrates that Foxp3-induced factors play a major role.

### Blocking of Nrp-1 Reduces the Number of Long Interactions Made by Treg Cells

**Figure 4:**
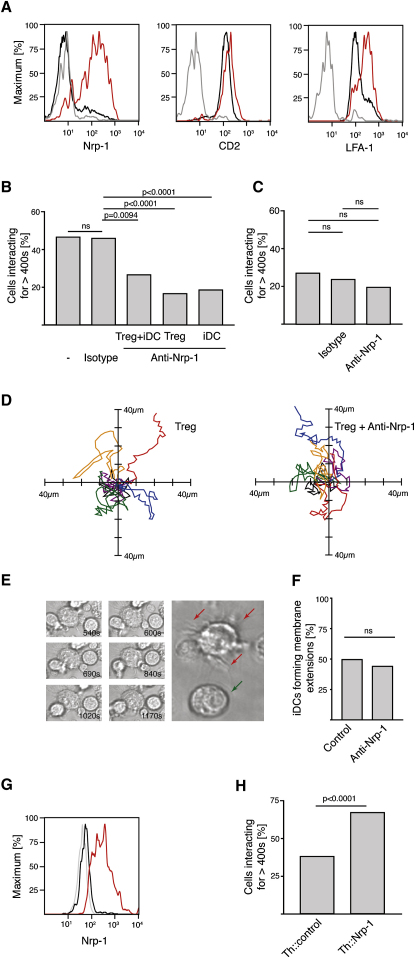
Nrp-1 on Treg Cells Promotes Long Interactions with iDCs (A) Overlays of CD4^+^CD25^−^ cells (black) and CD4^+^CD25^+^ cells (red) stained with the indicated antibodies (representative of four independent experiments) are shown together with the respective isotype control stains of CD4^+^CD25^−^ cells (gray). (B) Inhibition of long CD4^+^CD25^+^ (Treg) cell-iDC interactions by anti-Nrp-1 treatment. Percentage of Treg cells forming long interactions with iDCs in the absence of antibody (seven independent experiments, n = 234), in the presence of anti-Nrp-1 (Treg+iDC anti-Nrp-1; three independent experiments, n = 64) or isotype control (three independent experiments, n = 148), when only Treg cells were preincubated with anti-Nrp-1 (Treg anti-Nrp-1; five independent experiments, n = 165), and when only iDCs were preincubated with anti-Nrp-1 (iDC anti-Nrp-1 preincubation; two independent experiments, n = 70). (C) Anti-Nrp-1 treatment has no effect on long CD4^+^CD25^−^ (Th) cell-iDC interactions. Percentage of Th cells forming long interactions with iDCs in the absence of treatment (four independent experiments, n = 131) or in the presence of anti-Nrp-1 (two independent experiments, n = 51) or isotype control (two independent experiments, n = 63). (D) The presence of anti-Nrp-1 has no effect on the motility of CD4^+^CD25^+^ (Treg) cells on ICAM-1-coated plates. The origin of the traces (20 min recording) was centered on the x and y axis (three representative traces from two independent experiments in each case). (E and F) The presence of anti-Nrp-1 has no effect on the formation of membrane extensions by iDCs. (E) Snapshots of a representative iDC forming membrane extensions to grab T cells (images on the left). Two representative iDCs (large image on the right) are shown; one of them is not forming any membrane extensions (green arrow), and the other one is continuously forming new extensions (red arrows). (F) Percentage of iDCs forming membrane extensions in the absence (control; two independent experiments, n = 101) or presence of anti-Nrp-1 (anti-Nrp-1; two independent experiments, n = 109). (G and H) Ectopic expression of Nrp-1 in Th cells leads to an increase in long interactions with iDCs. (G) FACS analysis of Th cells (black), Th::Nrp-1 cells (red) stained with anti-Nrp-1, and unstained nontransduced Th cells (gray). (H) Percentage of Th::Nrp-1 (two independent experiments, n = 106) and Th::control cells (two independent experiments, n = 158) forming long interactions (>400 s) with iDCs. The relevant p values (Fischer's exact test) are given. ns stands for statistically not significant.

Expression of Foxp3 in Treg cells leads to a multitude of differences in their transcriptional program compared to Th cells. This raises the question of which of the differentially expressed molecule (or molecules) accounts for the differences in interaction dynamics. Differentially expressed adhesion or costimulatory molecules are likely candidates. Our review of the literature suggested Nrp-1 to be a good candidate. In combination with Plexins of the A subfamily, Nrp-1 is a receptor for class III semaphorins and a coreceptor for vascular endothelial growth factor (VEGF) in combination with Flt-1 (VEGF-receptor 1) and Kdr (VEGF-receptor 2) (Kruger et al., 2005; Staton et al., 2007). In the context of the immune system, it has been suggested to be involved in the initiation of immune responses by promoting DC-T cell contacts through homotypic interactions (Tordjman et al., 2002). Within the T cell lineage, Nrp-1 is preferentially expressed on Treg cells and can be induced by ectopic expression of Foxp3 in Th cells (Bruder et al., 2004). Indeed, we found that more than 80% of CD4^+^CD25^+^ cells expressed Nrp-1, whereas we could detect it on only few CD4^+^CD25^−^ cells (Figure 4A). This is in stark contrast to other molecules known to be involved in T cell-APC interactions, such as CD2 and LFA-1 (Dustin et al., 1997), expressed highly by both cell types (Figure 4A).

We therefore decided to examine whether Nrp-1 is responsible for the difference in interaction behavior between CD4^+^CD25^+^ Treg and CD4^+^CD25^−^ Th cells. Addition of anti-Nrp-1 led to a 50% decrease in the frequency of long interactions between iDCs and CD4^+^CD25^+^ Treg cells (Figure 4B; Movies S5 and S6). In contrast, the frequency of long interactions between CD4^+^CD25^−^ Th cells and iDCs did not significantly change (Figure 4C). To follow up on the suggestion that Nrp-1 promotes T cell-DC contacts through homotypic interactions (Tordjman et al., 2002), we investigated the expression of the Nrp-1 coreceptors previously associated with its function on immune cells *Kdr* (Mor et al., 2004), *Flt-1* (Oyama et al., 1998), and *PlexinA1* (Wong et al., 2003) in Treg cells and iDCs (Figure S2). RT-PCR revealed that *Flt-1* and *PlexinA1* were expressed in iDCs. None of these coreceptors could be detected at a substantial level in Treg cells. Further support for a possible homotypic interaction of Nrp-1 being responsible for promoting long interactions between Treg cells and iDCs comes from our finding that preincubation of either Treg cells or iDCs with anti-Nrp-1 is sufficient to block long interactions between the two cell types (Figure 4B). However, the molecular basis and physiological role of Nrp-1 homotypic interactions remain poorly understood, and we cannot exclude the possibility that other coreceptors and ligands of Nrp-1 are involved (Kruger et al., 2005; Staton et al., 2007).

To ensure that anti-Nrp-1 does not interfere with the motility of the cells, we assayed the migration behavior of CD4^+^CD25^+^ Treg cells on ICAM-1-coated plates in the presence or absence of anti-Nrp-1. Because the cells were not preactivated, very few of them moved and anti-Nrp-1 had no effect on those that did migrate (Figure 4D). This poses the question of how the T cells and iDCs come into contact in the first place. Although some of this can be attributed to active and passive movement of the T cells and iDCs, the grabbing of T cells by membrane extensions of iDCs (Figure 4E) is probably the most important component. Thus, we examined the effect of anti-Nrp-1 on the formation of membrane protrusions and found it to be negligible (Figure 4F). Our observation suggests that anti-Nrp-1 treatment directly interfered with interactions between Treg cells and iDCs, decreasing the frequency of long interactions to the level found for naive Th cells.

### Ectopic Expression of Nrp-1 in Th Cells Increases the Number of Long Interactions

To test whether Nrp-1 is sufficient to induce a difference in interaction behavior, we transduced CD4^+^CD25^−^ Th cells with retroviral vectors expressing either Nrp-1 (Th::Nrp-1) or a control gene (Th::control). Nrp-1 expression was verified by flow cytometry (Figure 4G), and the interaction behavior of the transduced cells was assessed as described above. We found that Th::Nrp-1 cells made significantly more long interactions with iDCs than Th::control cells (67% versus 38%; Figure 4H). Yet again, the qualitative difference of the interaction behavior of Th::Nrp-1 and Th::control cells with iDCs matched that of CD4^+^CD25^+^ Treg and CD4^+^CD25^−^ Th cells. We conclude that Nrp-1 expression is sufficient to account for at least part of the difference in interaction behavior.

### Classification of T Cell-iDC Contacts

**Figure 5:**
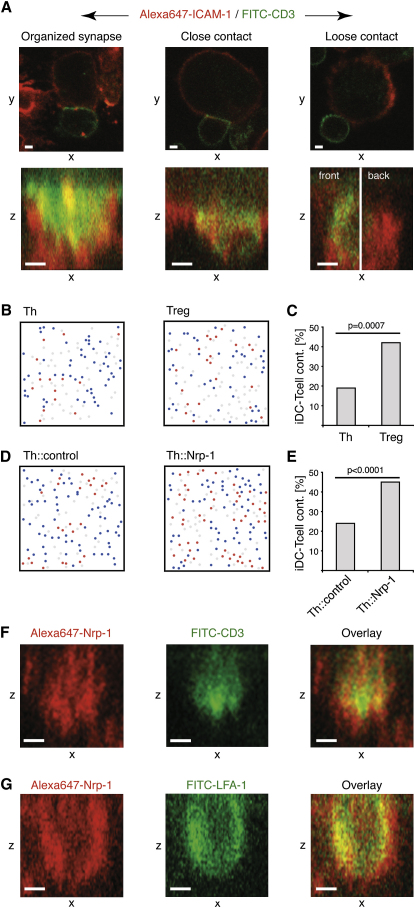
Analysis of Synapse Formation between T cells and iDCs (A) Images representative of an organized synapse, close contact, and loose contact on a single confocal section on the medial xy plane (top) or in a projection of zx images spanning 0.5 μm in the y direction in the area of the contact zone between the T cell and the iDC (bottom). In the case of the representative example of a loose contact, the projection of zx images is split in two halves spanning 0.5 μm in the y direction (front/back of the contact zone). (B–E) Frequency of contacts between T cells and iDCs after 20 min. Representative examples of the distribution of T cells in contact (red) and T cells not in contact (blue) with iDCs (gray) are shown. (B) CD4^+^CD25^+^ (Treg) and CD4^+^CD25^−^ (Th) cells and (D) Th::Nrp-1 and Th::control cells are shown. A summary of the frequency of (C) Treg (n = 95) and Th cells (n = 108) or (E) Th::Nrp-1 (n = 221) and Th::control (n = 221) cells in contact with iDCs (two independent experiments) is presented. (F and G) Localization of Nrp-1 in the synapse. CD4^+^CD25^+^ Treg cells from DO11.10 mice were cocultured with ova-loaded iDCs (100 μg/ml; 12 hr). Projections of zx images spanning 0.5 μm in the y direction of the contact zone between the Treg cell and the iDC are shown. White scale bars represent 2 μm. The relevant p values (Fischer's exact test) are given.

Next, we examined the relationship between long interactions and synapse formation between CD4^+^CD25^+^ Treg or CD4^+^CD25^−^ Th cells and iDCs. All iDC-T cell contacts found under transmission light after 25 min of coculture were scored according to the distribution of CD3 and ICAM-1 in the contact area. We distinguished three categories of iDC-T cell contacts: (1) organized synapses, (2) close contacts, and (3) loose contacts (Figure 5A). Organized synapses are marked by close apposition of the iDC-T cell membranes, polarization of CD3 on T cells toward the iDC-T cell interface, and segregation of CD3 and ICAM-1 into cSMAC and pSMAC (Monks et al., 1998). Contacts that showed close apposition of the iDC-T cell membranes but lacked a marked polarization of CD3 and clear segregation of CD3 and ICAM-1 were classified as close contacts. Contacts that exhibited neither a close apposition of the iDC-T cell membranes nor polarization of CD3 on the T cells were classified as loose contacts. An analysis of the synapses between CD4^+^CD25^−^ Th cells prepared from DO11.10 × SCID mice and iDCs preincubated with ova-loaded iDCs (100 μg/ml; 12 hr) further validated our definition of organized synapses (Figure S3).

### Treg Cells Form Synapses with iDCs More Frequently Than Naive Th Cells

Twelve percent of all contacts between WT Treg cells and iDCs were scored as organized synapses. The frequency of organized synapses formed between CD4^+^CD25^−^ Th cells and iDCs was slightly lower, at 9% (Table S1). However, the frequency of organized synapses relative to interactions does not take into account the fact that fewer interactions take part between Th cells and iDCs than between Treg cells and iDCs (Figures 5B and 5C) (19% versus 42%). Thus, the frequency of organized synapses relative to the number of cells provides a more accurate picture. Five percent of all Treg cells formed an organized synapse with an iDC opposed to 2% of all Th cells (Table S1).

### Ectopic Expression of Nrp-1 in Naive Th Cells Enhances Synapse Formation with iDCs

Because ectopic expression of Nrp-1 is sufficient to increase the frequency of long interactions formed by CD4^+^CD25^−^ Th cells, we investigated whether it has functional consequences by increasing the frequency of organized-synapse formation. Ectopic expression of Nrp-1 led to an increase in the number of contacts with iDCs (Figures 5D and 5E). Forty-five percent of Th::Nrp-1 cells had formed contacts with iDCs, in contrast to only 24% of Th::control cells. The frequency of synapses formed was similar for both Th::Nrp-1 (21%) and Th::control (22%) cells (Table S1). Taking the difference in the number of contacts into account, the frequency of synapses relative to the number of cells was about 2-fold higher in the case of Th::Nrp-1 cells (9%) than in the case of Th::control cells (5%) (Table S1). We conclude that ectopic expression of Nrp-1 in naive Th cells leads not only to an increase in the frequency of long interactions with iDCs, but also to an increase in the total number of synapses formed.

### Localization of Nrp-1 in the Synapse between Treg Cells and iDCs

The finding that Nrp-1 plays a role in synapse formation led us to examine whether Nrp-1 itself is localized in the synapse and whether it segregates into either the pSMAC or cSMAC region. We assessed the distribution of Nrp-1 in contacts between DO11.10 Treg cells and ova-loaded iDCs (100 μg/ml; 12 hr). To determine the pSMAC and cSMAC region of the synapse, we costained the contacts with LFA-1 and CD3. We found that Nrp-1 preferentially localized into the pSMAC region, although its exclusion from the cSMAC was not complete (Figures 5F and 5G).

### Ectopic Expression of Nrp-1 in Th Cells Increases Their Sensitivity to Antigen

**Figure 6:**
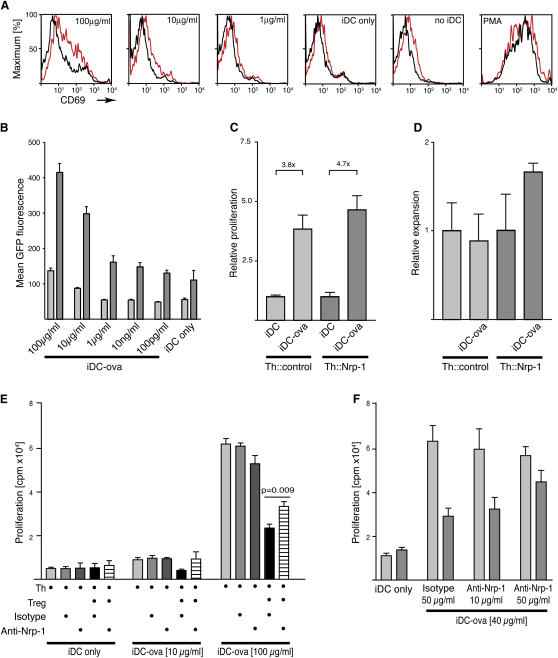
Nrp-1 Enhances T Cell Sensitivity to Antigen and Contributes to Treg Cell Suppression (A–C) Ectopic expression of Nrp-1 in CD4^+^CD25^−^ cells increases sensitivity to antigen ex vivo. Th::Nrp-1 or Th::control cells from DO11.10xWT mice were cocultured with iDCs (iDC only) or with ova-loaded iDCs (1–100 μg/ml; 12 hr). (A) CD69 expression on Th::Nrp-1 (red) and Th::control cells (black) after 24 hr of culture (representative example of two independent experiments). T cells cultured in the absence (no iDC) or presence of 50 ng/ml PMA stimulation (PMA) are shown as controls. (B) Th::Nrp-1 (dark bars) and Th::control cells (light bars) cotransduced with a retroviral vector carrying a NF-κB-driven GFP reporter gene are shown. GFP fluorescence was measured by flow cytometry after 24 hr of culture with the antigen-loaded iDCs. Error bars represent the standard error of the mean (SEM) from three independent experiments. (C) Relative proliferation of Th::Nrp-1 and Th::control cells after 56 hr of culture with untreated iDCs or ova-loaded iDCs (iDC-ova) (10 μg/ml; 12 hr), normalized to proliferation measured with untreated iDCs (pooled data from two independent experiments; n = 3), is shown. (D) Relative in vivo expansion of Th::Nrp-1 or Th::control cells from DO11.10xSCID mice in the spleens of nonimmunized mice or mice immunized with ova-loaded iDCs (100 μg/ml; 12 hr) (iDC-ova). The transduced cells were identified on the basis of the coexpression of GFP (two independent experiments, n = 6 for each group; p = 0.0359, unpaired t test). Error bars represent the SEM. (E and F) Anti-Nrp-1 treatment interferes with suppressive function of Treg cells. CD4^+^CD25^−^ (Th) cells were cocultured with CD4^+^CD25^+^ (Treg) cells (both prepared from DO11.10 mice) and either untreated iDCs or ova-loaded iDCs, in the presence or absence of anti-Nrp-1 or isotype control. Proliferation was determined by ^3^H thymidine incorporation. (E) Effect of anti-Nrp-1 treatment at different concentrations of antigen. CD4^+^CD25^−^ cells were cocultured with ova-loaded iDCs (indicated concentrations; 12 hr), in the presence or absence of CD4^+^CD25^+^ cells, with or without anti-Nrp-1 or isotype control (10 μg/ml) (pooled data from three independent experiments performed in duplicates; p = 0.009, unpaired t test). Error bars represent the SEM. (F) Dose-dependent effect of anti-Nrp-1 treatment. CD4^+^CD25^−^ cells were cocultured with (dark bars) or without (light bars) CD4^+^CD25^+^ cells, in the presence of the indicated amounts of anti-Nrp-1 or isotype control (n = 4).

Synapse formation between T cells and APCs is thought to depend on the presence of cognate antigen (Grakoui et al., 1999). However, ectopic expression of Nrp-1 in CD4^+^CD25^−^ Th cells leads to an increase in the number of synapses formed with iDCs. An explanation for this might be that in some cases, synapse formation fails, despite the T cell having the right TCR specificity, as a result of an insufficient abundance of a particular MHC class II-peptide complex. Ectopic expression of Nrp-1 leads to an increase in interaction time, which may allow the T cells more time to gather sufficient MHC class II-peptide complexes and/or associated signals to allow synapse formation. On a population level, this would result in the observed increase in the number of synapses formed. To test this hypothesis, we examined whether Nrp-1 increases the sensitivity of T cells toward their cognate antigens. We compared the response of Th::Nrp-1 and Th::control cells from DO11.10 mice to varying amounts of ova. Initially, we assessed the upregulation of CD69, which is commonly used to measure the activation status of T cells (Benvenuti et al., 2004). We optimized our transduction protocol so that the expression of CD69 remained close to background (Figure 6A; no iDC). Unspecific activation of the cells with PMA led to upregulation of CD69 on all cells irrespective of Nrp-1 expression (Figure 6A; PMA). Addition of iDCs only had a very minor effect (Figure 6A; iDC only). To compare the antigen sensitivity of the Th::Nrp-1 and Th::control cells, we titrated the antigen load of the iDCs by preincubating them with increasing amounts of ova. Our data showed that lower doses of cognate antigen were required to activate Th::Nrp-1 cells compared to Th::control cells (Figure 6A; 1–100 μg/ml). To substantiate our findings, we assessed the transcriptional activation of NF-κB, an event that follows TCR signaling (Sun et al., 2000). We cotransduced CD4^+^CD25^−^ Th cells from DO11.10 mice with a vector carrying a NF-κB-driven GFP as reporter gene and a vector carrying Nrp-1 (Figure S4 and Figure 6B). The cotransduction efficiency was approximately 2%. The activation of the cells transduced only with the reporter construct was compared to that of the cells transduced with both constructs. Addition of iDCs led to a small increase in baseline activation, which was more marked in the Nrp-1-transduced cells. In analogy to the experiment described above, we titrated the loading of the iDCs with ova. Yet again, we found that Nrp-1-transduced cells responded to smaller amounts of the cognate antigen (Figure 6B). Next, we addressed whether the Nrp-1-induced increase in sensitivity translates into an increase in proliferation under circumstances when the amount of antigen presented is limiting. Th::Nrp-1 and Th::control cells from DO11.10 were cocultured with ova-loaded iDCs (10 μg/ml; 12 hr). In the case of the Th::Nrp-1 cells, we observed a 4.7-fold increase in ^3^H-thymidine incorporation, whereas we only observed a 3.8-fold increase in the Th::control cells (Figure 6C). Together, these findings suggest that the expression of Nrp-1 makes the T cells more sensitive to antigen.

Under physiological conditions, iDC are thought to be poor inducers of immune responses in the absence of proinflammatory signals and appear to have a tolerogenic effect on the immune system (Steinman et al., 2003; Steinman et al., 2000). Thus, we investigated the effect of ectopic expression of Nrp-1 in naive Th cells in vivo. We injected Th::Nrp-1 or Th::control cells from DO11.10 × SCID mice into WT mice and immunized half of them with ova-loaded iDCs (100 μg/ml; 12 hr). After 7 days, we analyzed the number of transduced cells in the spleen of the immunized and nonimmunized animals. We observed a marked expansion of Th::Nrp-1 cells upon immunization (Figure 6D). In contrast, Th::control cells did not expand, but rather contracted slightly. This finding suggests that the presence or absence of Nrp-1 not only has consequences on the sensitivity of T cells to antigen, but also alters how they respond to iDCs in vivo.

### Nrp-1 Blocking Interferes with Antigen-Specific Suppression by Treg Cells

Under physiological conditions, Nrp-1 is expressed in Treg, but not naive Th, cells. We therefore investigated how Nrp-1 might contribute to Treg cell function. If the role of Nrp-1 is to give Treg cells a head start over naive Th cells under conditions in which antigen is limiting, blocking of Nrp-1 function should release the suppressive effect. To test this, we cocultured CD4^+^CD25^−^ and CD4^+^CD25^+^ cells from DO11.10 mice with iDCs loaded with varying amounts of antigen and assessed the effect of anti-Nrp-1 treatment on the suppression of Th cell proliferation. iDCs that were loaded with 10 μg/ml ova lead only to a modest proliferation of the Th cells, which could be entirely suppressed by addition of Treg cells (Figure 6E). Under these conditions, the presence of anti-Nrp-1 (10 μg/ml) completely abrogated the suppression of Th cell proliferation by Treg cells. When we increased the amount of antigen, we observed a marked increase in the proliferation of the Th cells; however, the suppression by Treg cells became incomplete. Nevertheless, we observed a significant decrease in suppressive activity when anti-Nrp-1 was added (Figure 6E). When we varied the amount of anti-Nrp-1 added while keeping the amount of antigen presented constant, we found that the effect of anti-Nrp-1 was dose dependent (Figure 6F). Our findings are consistent with our hypothesis that due to Nrp-1, Treg cells have a higher sensitivity to antigen and thus an advantage over naive Th cells with the same antigen specificity.

### Activation of DCs Increases the Frequency of Long Interactions with Naive Th Cells

**Figure 7:**
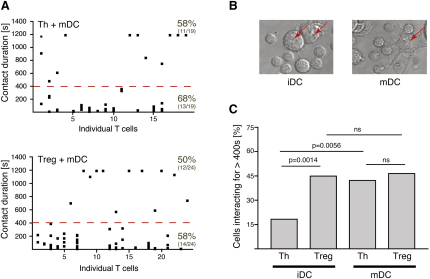
Activation of DCs Alters the Interaction with Naive Th Cells (A) Summaries of the interactions between CD4^+^CD25^+^ (Treg) or CD4^+^CD25^-^ (Th) cells and DCs preincubated in the presence of LPS (1 μg/ml; 12 hr) analyzed as outlined in Figure 1. (B) Representative examples of DCs (red arrows) that had been preincubated in the presence (mDC) or absence (iDC) of LPS. (C) Percentage of Treg or Th cells forming long interactions (>400 s) with DCs preincubated in the presence (mDC; two independent experiments, n = 43 for Treg and n = 44 for Th) or absence of LPS (iDC; two independent experiments, n = 49 for Treg and n = 82 for Th). The relevant p values (Fischer's exact test) are given. ns stands for statistically not significant.

Inflammatory stimuli, often referred to as “danger signals,” alter the interaction behavior of DCs with Th cells (Benvenuti et al., 2004). Mature DCs form more long interactions with Th cells than iDCs. We wondered whether activation of the DCs leads to a further enhancement of antigen sensitivity of Treg cells as is the case for Th cells or whether it has the opposite effect. CD4^+^CD25^+^ Treg or CD4^+^CD25^−^ Th cells were cocultured with bone-marrow-derived DCs that had been preincubated in the presence (mDCs; Figure 7A) or absence of lipopolysacharide (LPS). LPS stimulation caused characteristic changes in DC morphology (Benvenuti et al., 2004), such as an increase in cell size and formation of many large membrane extensions, quite distinct from those of iDCs (Figure 7B). Th cells formed more long interactions with mDCs (42.2%) than with iDCs (18.3%) (Figure 7C). However, LPS activation of DCs did not affect the frequency of long interactions between DCs and Treg cells. Thus, the activation of the DCs appears to level the playing field, as the frequency of long interactions with Th cells is raised to that of Treg cells.

## Discussion

The duration of the interaction between DCs and T cells not only is indicative of the outcome, but also might in itself be a crucial determinant. Short interactions between T cells and antigen-presenting cells are characteristic of the T cells scanning for antigen (Irvine et al., 2002; Miller et al., 2004a). Long interactions, however, only occur in the presence of cognate MHC-peptide complexes because TCR stimulation appears to be required to further stabilize the interaction, ultimately leading to synapse formation (Grakoui et al., 1999). We found that in the absence of exogenous antigen, Treg cells make twice as many long interactions with the iDCs than naive Th cells do. Although we cannot completely exclude the possibility that some of the peptides presented by the iDCs had exogenous origin, the majority of peptides presented are likely to have been of endogenous origin. Hence, we expected that at least part of the difference observed is due to a bias of the TCR repertoire of Treg cells toward self-antigens (Hsieh et al., 2004). However, ectopic expression of Foxp3 in Th cells had a profound effect on the frequency of long interactions, suggesting that lineage-specific determinants contribute to the interaction behavior of the cells. Indeed, our results demonstrate that Nrp-1, which is expressed on Treg cells but not naive Th cells and induced by ectopic expression of Foxp3 in the latter (Hill et al., 2007), can account for the difference in interaction behavior of the two cell types.

On a mechanistic level, it has been proposed that persistent TCR signals are required for the progression of T cell activation and that the barrier of rare MHC-peptide complexes is overcome by active concentration of the available complexes (Grakoui et al., 1999). In the context of limiting-antigen concentrations, a prolongation of the interaction time is likely to be of advantage to T cells seeking a signal because it provides more time to gather MHC-peptide complexes. Our results suggest that in some cases naive Th cells fail to form synapses despite expressing a TCR with the right specificity, because the concentration of the antigen presented is too low to lead to synapse formation. Indeed, we found that ectopic expression of Nrp-1 in Th cells leads to a higher sensitivity to their cognate antigen. We therefore propose that Treg cells have a higher sensitivity to antigen, because their expression of Nrp-1 allows them more time for the gathering of MHC class II-peptide complexes presented by iDCs.

iDCs are characterized by high endocytic activity and low T cell activation potential (Inaba et al., 2000; Steinman et al., 2003). They are thought to constantly sample the surrounding environment for pathogens (Banchereau and Steinman, 1998). In the absence of maturation stimuli in the form of proinflammatory signals, they cannot induce an immune response (Inaba et al., 2000; Steinman et al., 2003). They even appear to have a tolerogenic effect on the immune system, and it has been suggested that the uptake of dying cells by iDCs is critical for the maintenance of peripheral tolerance (Steinman et al., 2003; Steinman et al., 2000). Interestingly, we found that Th cells ectopically expressing Nrp-1 in contrast to control-transduced cells responded to antigen presented by iDCs in vivo. It appears that the presence of Nrp-1 on the T cells was sufficient to alter their response to antigen presented by iDCs in the absence of inflammatory stimuli.

In a situation in which both Th and Treg cells recognize the same antigen presented by iDCs, the Treg cells prevail and suppress the response. We propose that Nrp-1 expression on Treg cells gives them an advantage over naive Th cells in the absence of proinflammatory stimuli. Given that this favors Treg cell over Th cell activation, one might expect this to result in a suppression of immune responses in the absence of signals associated with pathogen invasion. However, upon exposure to “danger,” Treg cells lose this advantage. Any antigen recognized by the TCR repertoire of Treg cells, be it autoantigen or exogenous antigen, is likely to be subject to this scrutiny. This might be part of the explanation for why autoimmune responses can be readily induced by immunization with self-antigen in the presence of adjuvant and why infections pose a high risk factor during pregnancy (Rovere-Querini et al., 2005; Trowsdale and Betz, 2006).

## Experimental Procedures

### Animals and Cell Preparations

Animal care was performed by expert technicians in compliance with relevant laws and institutional guidelines. All mice were maintained under specific pathogen-free conditions. Lymphocytes were prepared from spleens with Lympholyte M (Cedarlane Laboratories). CD4^+^ cells were isolated by depletion with CD11b, CD11c, GR1, CD19, and CD8 antibodies (BD) via an autoMACS (Miltenyi). For preparation of CD4^+^CD25^−^ subpopulations, anti-CD25 (BD) was added. CD4^+^CD25^+^ were purified from CD4^+^ cells with the same anti-CD25. Cell purity was checked by flow cytometry. Hematopoietic progenitor cells were prepared by depletion of bone-marrow cells with CD16/32, CD11b, CD4, GR1, CD19, and CD8 antibodies (BD). Cells were cultured in RPMI (10% FCS, 50 ng/ml GM-CSF, 20 ng/ml IL-4 [Peprotech EC]) for 7 days. For obtaining mDCs, cells were incubated for > 12 hr with 1 μg/ml LPS (Sigma-Aldrich) and washed prior to use.

### Time-Lapse Video Microscopy

iDCs (5 × 10^4^) and T cells (1 × 10^5^) were observed in RPMI/20 mM HEPES on Lab-Tec chambered coverslips (0.8 cm^2^) (Nalge Nunc International) on a BioRad Radiance 2000 confocal microscope with heated stage (37°C) (BioRad/Zeiss). After 5 min, DIC images were acquired for 20 min (10 s intervals; 40× 1.3 numerical aperture). Cell interactions were analyzed with Adobe AfterEffects 6.5 Pro. In blocking studies, T cells and iDCs were first preincubated (10 min; 4°C) with 2 μg/ml anti-CD16/32 (eBioscience) (Fc receptor blocking) and then 10 μg/ml of anti-Nrp-1 (H-286) or isotype control (both Santa Cruz) or anti-LFA-1 (M17/4, eBioscience) or anti-ICAM-1 (3E2, BD) (30 min; 4°C) before analysis of interactions in the continuous presence of the antibodies at 37°C. For cell-specific blocking, either CD4^+^CD25^+^ cells or iDCs were preincubated and blocked as above, but the cells were washed before coculture, which was performed in the absence of the antibodies. TCR signaling was inhibited by preincubation (1 hr; 10 ng/ml; 37°C) of T cells with CsA or rapamycin (both Sigma-Aldrich). For motility assessment, the T cells were allowed to settle for 30 min, in the presence or absence of anti-Nrp-1 (H-286) or isotype control (both Santa Cruz, California), on ICAM-1 (3 μg/ml; R&D systems)-coated chambered coverslips and then observed for 20 min.

### Retroviral Transduction

Retroviral constructs for the delivery of Foxp3 (m6ph[Foxp3] and m6p8[Foxp3]), Nrp-1 (m6pt[Nrp-1], m6ph[Nrp-1], and m6pg[Nrp-1]), control genes (m6ph[control], m6p8[control], m6pt[control], and m6pg[control]), and NF-κB-driven GFP-reporter construct m7p8 (κB > GFP) are shown in Figures S1A and S4. Constructs differ in the coexpressed marker genes GFP, GPI-linked rat CD8α (ratCD8), histidinol-dehydrogenase (HisD), and mouse Thy1.1 used to identify or select transduced cells. 293ET cells were cotransfected with pCl-Eco packaging plasmid and either of the constructs above. After 42 hr, the supernatant was filtered and used immediately. Freshly purified CD4^+^CD25^−^ T cells were preactivated for 36 hr on 1 μg/ml plate-bound anti-CD3ɛ (145-2C11; BD) and 5 ng/ml murine IL-2 (Peprotech EC) and transduced with a 1:3 dilution of viral supernatant supplemented with 6 μg/ml protamine sulfate (Sigma-Aldrich), followed by centrifugation for 2 hr at 1800 rpm. Transduced cells were cultured for 3 days in RPMI/IL-2 (5 ng/ml). Selection of transduced cells was performed prior to time-lapse video microscopy, measurement of proliferation, or RT-PCR analysis. For HisD-selection (24 hr after transduction), cells were cultured for 2 days in the presence of 2.5 mM histidinol (Sigma-Aldrich). Viable cells were purified with Lympholyte M. Alternatively, transduced cells were selected with either anti-ratCD8a or anti-Thy1.1 via an autoMACS. For experiments requiring subsequent activation measurements (CD69, NF-κB activation reporter, proliferation experiments ex vivo and in vivo), an alternative transduction protocol was used. Cells were transduced after 18 hr of activation (conditions as above). Four hours after transduction, cells were transferred into RPMI/ IL-2 (5 ng/ml) for 3–4 days.

### Flow-Cytometry Analysis, T Cell Stimulation, and immunohistochemistry

A list of the antibodies used can be found in the Supplemental Data. iDCs (2 × 10^4^) and T cells (4 × 10^4^) were incubated (37°C; 25 min) on poly-L-lysine (Sigma-Aldrich)-coated multispot microscope slides (0.283 cm^2^; C.A. Hendley-Essex) and then fixed with 4% para-formaldehyde (Electron Microscopy Sciences) (20°C; 15 min) and then permeabilized with 0.02% saponin (Sigma-Aldrich) (in PBS; 10 min). Blocking in 5% FCS (PerBio) was followed by 45 min incubation with the primary antibody. After washing, the slides were blocked with 3% donkey serum (Jackson Laboratories) and stained with the secondary antibody for 45 min. The slides were sealed with Vectashield (H-1000, Vector Laboratories) and used for imaging. Z axis stacks (0.2 μm steps) of xy DIC and fluorescent images were collected (60× 1.3 numerical aperture) and analyzed with the Volocity (Improvision). All images were blinded prior to analysis and scoring.

### Measurement of Antigen Sensitivity

Fifty thousand selected m6pt[control] or m6pt[Nrp-1] transduced CD4^+^CD25^−^ cells from DO11.10 mice were plated in flat-bottom plates (Corning) together with 2.5 × 10^4^ iDCs that had been preincubated for 12 hr with ova (100 pg/ml–100 μg/ml, Sigma-Aldrich). After 36 hr, the cells were pulsed with 1 μCi [^3^H]thymidine (Amersham) for 18 hr, harvested, and analyzed with a TopCount microplate scintillation counter (Packard BioScience). For activation measurement based on upregulation of CD69, the transduced cells were cocultured as above. However, in this case, transduced and nontransduced cells were distinguished on the basis of the coexpression of Thy1.1 at the time of analysis. When NF-κB activation was measured, the cells were cotransduced with m7p8[κB > GFP] and m6pt[Nrp-1] (Figure S4). The GFP expression in m7p8[κB > GFP], m6pt[Nrp-1] double-transduced ratCD8^+^ Thy1.1^+^ cells was compared to that in m7p8[κB > GFP] single-transduced ratCD8^+^ cells after 24 hr.

### T Cell Suppression Assays

CD4^+^CD25^−^ cells (5 × 10^4^) were labeled (15 min, 37°C) with 5 μM CFSE (Invitrogen) and plated in U-bottom plates (Corning) with irradiated (3000 rad) splenocytes (5 × 10^4^) and CD4^+^CD25^+^ cells (5 × 10^4^) transduced with either m6ph[Foxp3] or m6ph[control]. Proliferation of the cells was after 3 days by flow cytometry. Proliferation of Th::Foxp3 or Th::control cells was assessed by measuring cell counts relative to CaliBRITE-beads (BD). The effect of anti-Nrp-1 on Treg cell suppression was assessed by coculturing ova-loaded (10–100 μg/ml; 12 hr), irradiated (3000 rad) iDCs (5 × 10^3^) preincubated with anti-CD16/32 (2 μg/ml; 4°C, 15 min) with CD4^+^CD25^+^(2.5 × 10^4^) and CD4^+^CD25^−^ cells (2.5 × 10^4^) from DO11.10 mice in the presence of anti-Nrp-1 or isotype control (both R&D Systems) in U-bottom plates. Proliferation of the cells was measured on the basis of ^3^H-thymidine (as above).

### In Vivo T Cell Expansion

CD4^+^CD25^−^ cells from DO11.10 × SCID mice were transduced with either m6pg[Foxp3] or m6pg[control] and injected after 3–4 days of resting intravenously (i.v.) into Balb/c mice. The number of total T cells transferred (containing both transduced and nontransduced cells) was kept constant at 10^6^ cells/mouse. The transduction efficiency was 15%–40%. Twenty-four hours later, the mice were immunized by i.v. injection of 10^5^ iDCs that had been preincubated with 100 μg/ml ova for 12 hr. Proliferation of transduced cells was analyzed 7 days after immunization by flow cytometry.

### Quantitative RT-PCR

cDNA was synthesized with SuperscriptII RT (Invitrogen), with random hexamer primers followed by amplification via either Taqman universal PCR master mix or SYBR Green PCR master mix (Applied Biosystems). A list of primers can be found in the Supplemental Data.

### Statistical Methods

Statistical analysis was done with Prism3 (GraphPad). The p values were calculated with a two-tailed Fisher's exact test (95% confidence intervals) or with a two-tailed unpaired t test (95% confidence intervals).

## Supplemental Data

Document S1. Supplemental Experimental Procedures, One Table, and Four Figures

Movie S1. Interactions of iDCs with Th Cells All the movies are 60-fold accelerated time-lapse videos of the interactions between iDCs and T cells. Differential interference contrast (DIC) images were collected every 10 s for 20 min with a 40× 1.3 numerical aperture objective. Blocking antibodies were added as indicated. The iDC and T cells are in the vast majority of cases clearly identifiable on the basis of size and morphology. All movies shown are taken from a representative cropped area of the 6.6-fold-larger analyzed field of vision. In all cases, the experiments were repeated multiple times, and all T cells within the field of vision that had made a contact with an iDC were included in the analysis.

Movie S2. Interactions of iDCs with Treg Cells

Movie S3. Interactions of iDCs Prepared from *MHCII*^−/−^ Mice with Treg Cells

Movie S4. Interactions of iDCs with Treg Cells in the Presence of anti-ICAM-1

Movie S5. Interactions of iDCs with Treg Cells in the Presence of anti-Nrp-1

Movie S6. Interactions of iDCs with Treg Cells in the Presence of an Isotype Control
