# Supplementary material for: Neuropilin-1 Expression on Regulatory T Cells Enhances Their Interactions with Dendritic Cells during Antigen Recognition
Source: Immunity. 2008 Mar 14;28(3):402–13. doi: 10.1016/j.immuni.2008.01.012 (PMC2726439; doi:10.1016/j.immuni.2008.01.012)
Supplement: Document S1. Supplemental Experimental Procedures, One Table, and Four Figures [file mmc1.pdf]

Immunity, Volume 28

Supplemental Data

Neuropilin-1 Expression on Regulatory T Cells Enhances Their Interactions with Dendritic Cells during Antigen Recognition

Milka Sarris, Kristian G. Andersen, Felix Randow, Luzia Mayr, and Alexander G. Betz

Supplemental Experimental Procedures

Additional Information Regarding Flow-Cytometry Analysis, T Cell Stimulation, and Immunohistochemistry

The following antibodies were used according to the manufacturer's instructions: PE-conjugated rat CD8a (OX-3; BD), FITC or PE-conjugated CD25 (7D4; BD), PE-conjugated CD69 (H1.2F3; BD), PE-conjugated Foxp3 (FJK-16s; eBioscience), PE-conjugated GITR (108619; R&D Systems), FITC-conjugated CD2 (eBioscience), FITC-conjugated rat IgG2a (eBioscience), FITC-conjugated rat IgG2b (eBioscience), PE-conjugated Lag3 (C9B7W; Santa Cruz Biotechnology), PE-Cy5-conjugated CD4 (L3T4, BD), APC or biotin-conjugated Thy1.1 (HIS51; eBioscience), purified, or FITC-conjugated CD3ε (145-2C11; BD), FITC conjugated LFA-1 (M17/4, eBioscience), goat anti-ICAM-1 (R&D Systems), goat anti-Nrp-1 (R&D systems), FITC-conjugated donkey anti-goat IgG (Jackson Immunoresearch) and Alexa-647 conjugated donkey anti-goat IgG (Molecular Probes).

Additional Information Regarding Quantitative RT-PCR

The following primers and probes were used for quantitative RT-PCR:

Foxp3:	5’-CCC AGG AAA GAC AGC AAC CTT-3’
	5’-TTC TCA CAA CCA GGC CAC TTG-3’
	5’-FAM-ATC CTA CCC ACT GCT GGC AAA TGG AGT C-3’;
Hprt:	5’-TTA AGC AGT ACA GCC CCA AAA TG-3'
	5'-CAA ACT TGT CTG GAA TTT CAA ATC C-3'
	5'-VIC-CCT TTT CAC CAG CAA GCT TGC AAC CTT A-TAMRA-3'
Flt-1:	5'-AGC AGG CCA GAC TCT CTT TCT CAA-3’
	5'-TTT GTC CTC CTG GCT CAC GGT-3’
Kdr:	5'-ACG TCG ACA TAG CCT CCA CTG TTT-3’
	5'-TTC TCG GTG ATG TAC ACG ATG CCA-3’
PlexinA1:	5-AAT CCT GCT ACC GTG GAG AAG GAA AG-3’
	5'-AGA AGT CGT CAT CAA TCT GCA GGG-3’
Nrp-1:	5'-AGC AAG CGC AAG GCT AAG TC-3’;
	5'-ATC CTG ATG AAC CTT GTG GAG AGA-3’

Table S1. Frequency of Synapse Formation among Interactions of iDCs with Treg and Naive Th Cells, as well as Th::[Nrp-1] and Th::[control] Cells

	Relative to Interactions [%]				Relative to Cells [%]			
	Treg n=66	Th n=43	Th::Nrp-1 n=38	Th::control n=38	Treg	Th	Th::Nrp-1	Th::control
organized synapses	12	9	21	22	5	2	9	5
close contacts	32	37	37	44	13	7	17	11
loose contacts	56	54	42	34	24	10	19	8
no contact					58	81	55	76

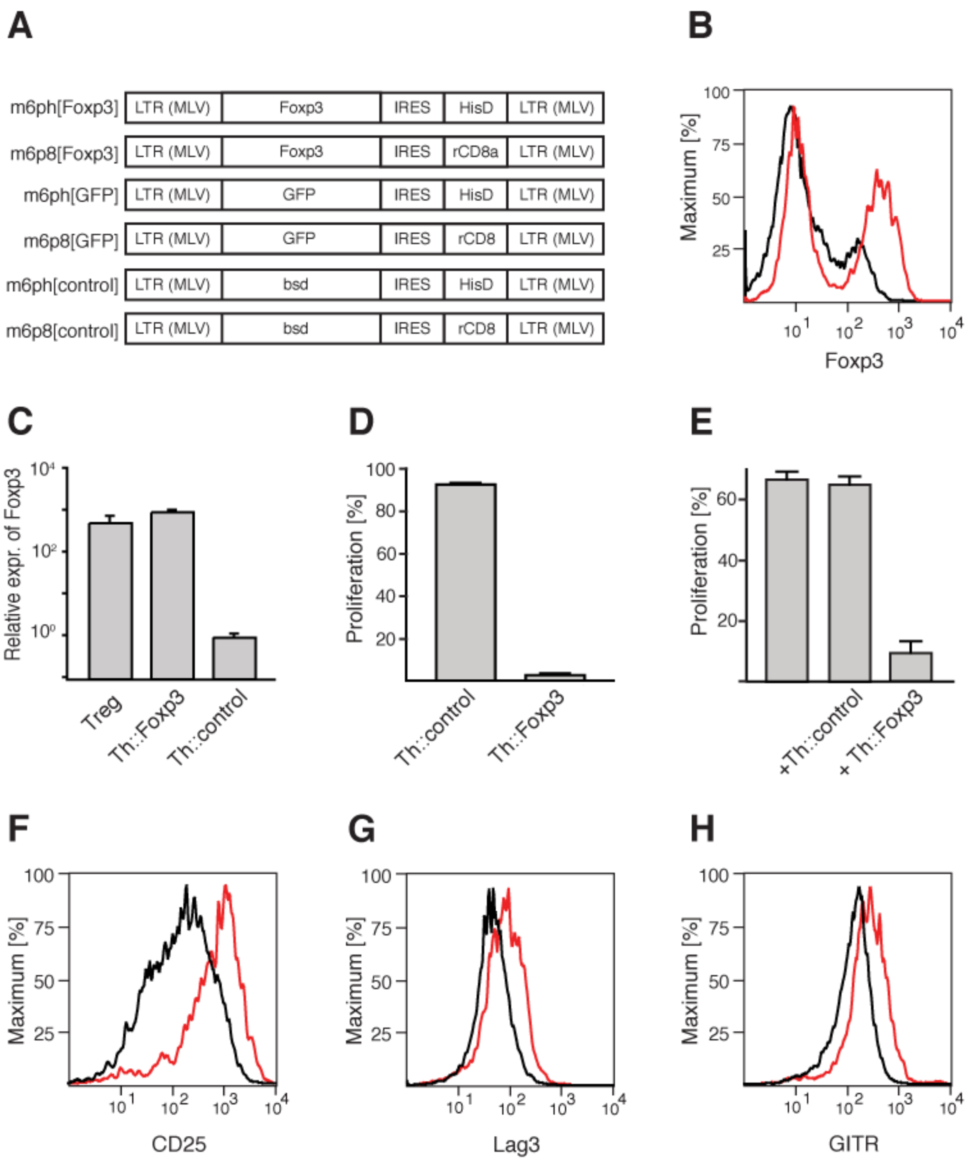

Figure S1. Foxp3 Transduction of Th Cells

(A) Foxp3 was amplified from Balb/c cDNA and cloned into the retroviral vectors m6ph and m6p8 carrying an IRES Histidinol-Dehydrogenase (HisD) or IRES GPI-linked ratCD8 casette. Equivalent constructs in which the Foxp3 was replaced by GFP or blasticidine-S-deaminase (bsd) were used as controls.

(B) FACS analysis of Foxp3 expression in Th::Foxp3 cells (red) and freshly isolated $CD4^+$ cells (black).

(C) Foxp3 mRNA levels (normalized to Hprt levels) in $CD4^+CD25^+$ cells and Th::Foxp3 or Th::control (bsd) cells.

(D) Proliferation of Th::Foxp3 or Th::control (GFP). The bars show the relative numbers of proliferated cells compared to an internal standard (CaliBRITE beads).

(E) Assessment of suppressive activity of Th::Foxp3 or Th::control (bsd). The percentage of cells that have proliferated after 3 days is shown.

(F-H) Flow-cytometric analysis of Th::Foxp3 cells. (F) CD25, (G) Lag3 and (H) GITR expression in Th::Foxp3 (red) or Th::control (bsd) (black) cells.

All error bars show the standard error of the mean from triplicates.

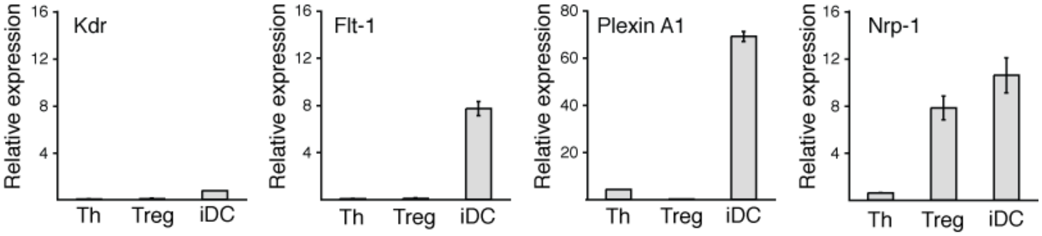

Figure S2. Comparative Analysis of the Expression of Nrp-1 and Its Coreceptors in Th Cells, Treg Cells, and iDCs

Relative expression of Kdr (VEGF-R2), Flt-1 (VEGF-R1), Plexin A1 and Nrp-1 in $CD4^{+}CD25^{-}$ Th cells, $CD4^{+}CD25^{+}$ Treg cells, and iDCs. mRNA levels were measured by quantitative real-time PCR and normalized to Hprt. All error bars show the standard error of the mean from duplicates.

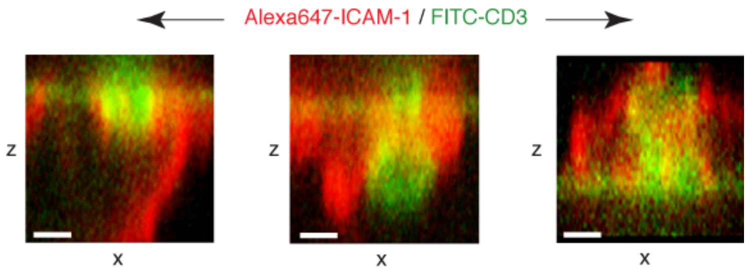

Figure S3. Representative Examples of Organized Synapses

Projection of zx confocal images spanning 0.5 µm in the y direction in the area of the contact zones between DO11.10 x SCID Th cells and ova-loaded [100µg/ml; 12 hr] iDCs. White scale bars on the images correspond to 2 µm.

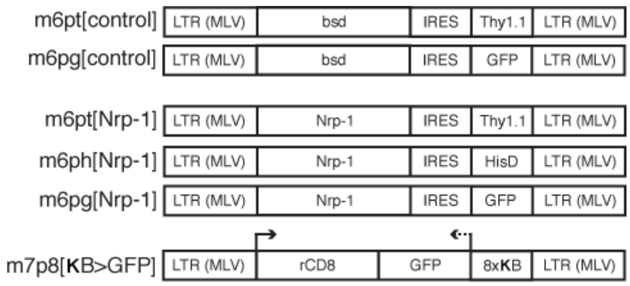

Figure S4. Retroviral Constructs

Nrp-1 was amplified from the image clone #6409596 and cloned into the retroviral vectors m6pt, m6ph and m6pg carrying an IRES mouse Thy1.1 marker, an IRES Histidinol-Dehydrogenase (HisD) or IRES GFP casette. Equivalent constructs in which the Nrp-1 was replaced by GFP (see Figure S1) or blasticidine-S-deaminase (bsd) were used as controls. The m7p8[κB>GFP] is a retroviral vector on which the constitutive expression of a GPI-linked rat CD8 is driven in the 'forward' direction and the NF-κB-inducible expression (8 concatenated κB-sites) of GFP in the 'reverse' direction
